# An outbreak of cutaneous abscesses caused by Panton-Valentine leukocidin-producing methicillin-susceptible *Staphylococcus aureus* among gold mine workers, South Africa, November 2017 to March 2018

**DOI:** 10.1186/s12879-020-05352-5

**Published:** 2020-08-24

**Authors:** Husna Ismail, Nelesh P. Govender, Ashika Singh-Moodley, Erika van Schalkwyk, Liliwe Shuping, Itumeleng Moema, Gal Feller, Ruth Mogokotleng, Wilhelmina Strasheim, Michelle Lowe, Ruth Mpembe, Serisha Naicker, Tsidiso G. Maphanga, Cecilia De Abreu, Farzana Ismail, Nazir Ismail, Mushal Allam, Arshad Ismail, Tanusha Singh, Onnicah Matuka, Thabang Duba, Olga Perovic

**Affiliations:** 1 Centre for Healthcare-Associated Infections, Antimicrobial Resistance and Mycoses, National Institute for Communicable Diseases, a division of the National Health Laboratory Service, 1 Modderfontein Road, Sandringham, Johannesburg, 2131 South Africa; 2 Faculty of Health Sciences, University of Witwatersrand, 7 York Road, Parktown, Johannesburg, 2193 South Africa; 3 South African Field Epidemiology Training Programme, National Institute for Communicable Diseases, a division of the National Health Laboratory Service, 1 Modderfontein Road, Sandringham, Johannesburg, 2131 South Africa; 4 Centre for Tuberculosis, National Institute for Communicable Diseases, a division of the National Health Laboratory Service, 1 Modderfontein Road, Sandringham, Johannesburg, 2131 South Africa; 5 Sequencing Core Facility, National Institute for Communicable Diseases, a division of the National Health Laboratory Service, 1 Modderfontein Road, Sandringham, Johannesburg, 2131 South Africa; 6 Immunology and Microbiology, National Institute for Occupational Health, a division of the National Health Laboratory Service, 25 Hospital Street, Constitution Hill, Johannesburg, 2000 South Africa

**Keywords:** Outbreak, Panton-valentine leukocidin, Methicillin-susceptible *Staphylococcus aureus*, Skin infection, Miners

## Abstract

**Background:**

We aimed to describe an outbreak of cutaneous abscesses caused by Panton-Valentine leukocidin (PVL)-producing methicillin-susceptible *Staphylococcus aureus* (MSSA) among gold mine workers.

**Methods:**

In February 2018, we retrospectively reviewed a random sample of 50 medical records from 243 cases and conducted face-to-face interviews using a structured questionnaire. Pus aspirates were sent to the National Institute for Communicable Diseases from prospectively-identified cases (November 2017–March 2018). Nasopharyngeal swabs were collected during a colonisation survey in February 2018. *Staphylococcus aureus* isolates were screened with a conventional PCR for *lukS/F*-PV. Pulsed-field gel electrophoresis (PFGE) was performed to determine the genetic relatedness among the isolates. A sample of isolates were selected for whole genome sequencing (WGS). We conducted an assessment on biological risks associated with mining activities.

**Results:**

From January 2017 to February 2018, 10% (350/3582) of mine workers sought care for cutaneous abscesses. Forty-seven medical files were available for review, 96% were male (*n* = 45) with a mean age of 43 years (SD = 7). About 52% (24/46) were involved in stoping and 28% (13/47) worked on a particular level. We cultured *S. aureus* from 79% (30/38) of cases with a submitted specimen and 14% (12/83) from colonisation swabs. All isolates were susceptible to cloxacillin. Seventy-one percent of *S. aureus* isolates (30/42) were PVL-PCR-positive. Six PFGE clusters were identified, 57% (21/37) were closely related. WGS analysis found nine different sequence types. PFGE and WGS analysis showed more than one cluster of *S. aureus* infections involving closely related isolates. Test reports for feed and product water of the mine showed that total plate counts were above the limits of 1000 cfu/ml, coliform counts > 10 cfu/100 ml and presence of faecal coliforms. Best practices were poorly implemented as some mine workers washed protective clothing with untreated water and hung them for drying at the underground surface.

**Conclusions:**

PVL-producing MSSA caused an outbreak of cutaneous abscesses among underground workers at a gold mining company. To our knowledge, no other outbreaks of PVL-producing *S. aureus* involving skin and soft tissue infections have been reported in mining facilities in South Africa. We recommend that worker awareness of infection prevention and control practices be strengthened.

## Background

*Staphylococcus aureus* is a Gram-positive bacterium that gives rise to a variety of infections, which include bloodstream, gastrointestinal, respiratory and skin and soft tissue infections (SSTIs) [1, 2]. *Staphylococcus aureus* forms part of the normal microflora of humans and approximately 20–30% of adult populations are colonized at any given time [3]. *Staphylococcus aureus* infections are common in both hospital and community settings [1]. Although a number of antimicrobial agents are used to treat staphylococcal infections, treatment remains a challenge due to antimicrobial resistance, particularly in healthcare settings [1, 2]. *Staphylococcus aureus* produces and expresses a range of exoproteins including the cytotoxin, Panton-Valentine leukocidin (PVL) [4]. The PVL is a pore-forming leukocidal toxin encoded by the *lukS-PV* and *lukF-PV* genes, which is known to cause leukocyte destruction and tissue necrosis [3–5].

In November 2017, the National Institute for Communicable Diseases (NICD) was alerted by a gold mining company in Gauteng province, South Africa of a large number of mine workers seeking care at their on-site occupational health clinic with skin infections, mostly cutaneous abscesses. From January through to September 2017, 243 mine workers had attended the occupational health clinic for the treatment of cutaneous abscesses. No deaths were reported. Following a stakeholders’ meeting involving management of the mining company, healthcare practitioners, occupational health practitioners and environmental health practitioners, we initiated epidemiological and laboratory investigations. In this study, we aimed to describe this outbreak investigation of cutaneous abscesses among gold mine workers and to characterise these PVL-producing methicillin-susceptible *S. aureus* (MSSA) isolates and relatedness with each other by using molecular methods.

## Methods

### Epidemiological investigations

#### Medical record review

On 6 February 2018, we retrospectively reviewed a random sample of medical records from 243 cases to describe the clinical management and treatment of mine workers with cutaneous abscesses. A standardised case report form was used to collect demographic, medical history and clinical data. A case was defined as any person employed at the mine who sought care at the occupational health clinic for cutaneous abscesses from 1 January through to 30 September 2017. A recurrent episode was defined when a cutaneous abscess recurred on a different part of the body or following the resolution of a previous episode.

#### Interviews

On 21 February 2018, we conducted face-to-face interviews using a structured questionnaire (Questionnaire S1). An eligible participant for an interview was defined as any person aged 18 years or older, engaged in underground mine work and employed for at least 1 month (as of the date of interview) at the gold mine. Written informed consent was obtained for interviews. An information sheet was provided to potential participants. We collected data on demographic characteristics, past medical history, exposure history and history of cutaneous abscesses. Eligible participants with cutaneous abscesses at the time of interview were examined by a medical doctor and a specimen (either pus aspirate and/or skin scrapings) was collected and submitted to the NICD for diagnostic testing. Written informed consent was obtained for specimen collection.

### Laboratory investigations

#### Clinical specimens

From 17 November 2017 to 13 March 2018, clinic personnel at the on-site occupational health clinic obtained pus aspirates from mine workers with cutaneous abscesses and submitted specimens to the NICD.

#### Nasopharyngeal carriage of *S. aureus*

Colonisation with *S. aureus* was determined by collecting posterior nasopharyngeal swabs from mine workers working during the morning shift on 21 February 2018. Written informed consent was obtained for specimen collection. Nasopharyngeal swabs were tested at the NICD.

#### Microbiological assays

Pus aspirates, skin scrapings and nasopharyngeal swabs were cultured as per standard microbiological procedures [6]. Bacterial colonies were sub-cultured onto fresh 5% horse blood agar plates (Diagnostic Media Products (DMP), National Health Laboratory Service (NHLS), South Africa) and identified to species level using a matrix assisted laser desorption ionization-time of flight mass spectrometer (MALDI-TOF) (Bruker Daltonik, Bremen, Germany). Antimicrobial susceptibility testing (AST) was performed on bacterial colonies using the Gram-positive panel PM33 on the MicroScan Walkaway system (Beckman Coulter, Inc., Atlanta, USA). Minimum inhibitory concentrations (MIC) were according to Clinical and Laboratory Standards Institute (CLSI) breakpoint recommendations [7].

#### Molecular characterisation of PVL-producing *S. aureus*

Genomic DNA from pure bacterial cultures grown on 5% horse blood agar plates (DMP, NHLS) was extracted using a crude boiling method and was then used as a template for polymerase chain reaction (PCR) amplification. Bacterial isolates were screened for the *luk*S/F-PV gene by a conventional PCR assay using the G-Storm thermal cycler (Somerton Biotechnology Centre, Somerton, UK), the Qiagen Multiplex PCR kit (Qiagen, Nordrhein-Westfalen, Germany) and previously published primers [8]. *Staphylococcus aureus* ATCC49775 was used as a positive control.

#### Pulsed-field gel electrophoresis

The genetic relatedness of the isolates was determined by pulsed-field gel electrophoresis (PFGE). Bacterial isolates cultured from pus aspirates, skin scrapings and nasopharyngeal swabs were selected based on the period of specimen collection. CDC-H9812 *Salmonella enterica* serotype Braenderup was included as a reference standard. PFGE for *S. aureus* was performed according to a published protocol [9]. PFGE banding patterns were analysed using BioNumerics (version 6.5) software (Applied Maths, Sint-Martens-Latem, Belgium). A dendrogram was created and compared using the unweighted pair group method with arithmetic mean analysis. Cluster analysis was conducted using a dice coefficient with both an optimization value and tolerance factor of 1.5%.

#### Whole-genome sequencing analysis

To supplement PFGE, whole-genome sequencing (WGS) analysis was performed for a sample of isolates. *Staphylococcus aureus* isolates were selected based on specimen type, AST and PFGE banding patterns. Bacterial isolates were grown in brain heart infusion (BHI) broth (DMP, NHLS) and genomic DNA from each isolate was extracted using the QIAamp DNA minikit (Qiagen, Germany). Samples were prepared using the Nextera XT library prep kit (Illumina, Inc., California, USA) and the MiSeq platform (Illumina) was used to carry out 2 × 300 base pair sequencing with 100x coverage. Raw sequencing data were trimmed and the trimmed reads were assembled on the De Novo Assembly Tool using the CLC Genomics Workbench Software version 11 (Qiagen, Germany). We used assembled genome data to identify isolates to species level and to characterise and compare these to genome assembly data of two *S. aureus* isolates, one hospital-associated (9336 HA) and one community-associated (9588 CA), obtained from the NICD/GERMS antimicrobial resistance surveillance study in 2015. The assembled genome data were evaluated using the Nullarbor pipeline and included: species identification; multi locus sequence typing (MLST) and identification of antimicrobial-resistant genes and virulence genes [10]. The *spa* types were determined using a web-based tool: Center for Genomic Epidemiology http://genomicepidemiology.org/. The assembled files were submitted to the National Center for Biotechnology Information GenBank and are available under BioProject numbers: PRJNA560164 and PRJNA548666.

### Data analysis

Data were analysed using Stata version 14.2 (StataCorp LP, College Station, Texas, USA). Categorical data were expressed as frequencies, percentages and/or presented graphically. Characteristics of mine workers with and without cutaneous abscesses were evaluated using appropriate statistical measures (for example, a χ2 test and/or Fisher’s exact test were used to compare categorical variables). Nasopharyngeal colonisation was defined as *S. aureus* cultured from a nasopharyngeal swab. The nasopharyngeal colonisation rate of *S. aureus* was calculated by dividing the number of nasopharyngeal swab specimens that cultured *S. aureus* by the total number of nasopharyngeal swab specimens taken.

### Bio-risk assessment

We conducted an assessment on biological risks associated with mining activities to determine possible exposure sources for both surface and underground levels. Data were gathered by visual inspection and interviewing employees and management to obtain information on specific job tasks, work practices, engineering controls, water supply and personal protective clothing. The assessed areas included the male and female change rooms, laundry, occupational health clinic and the underground stope and tunnel. A Q-trak indoor air quality meter (dry bulb) and probe, model SN 7575 (TSI Instruments Ltd., UK) was used to measure ambient temperature in °C, percentage relative humidity and carbon dioxide simultaneously at underground sites on the day of risk assessment.

## Results

**Fig. 1:**
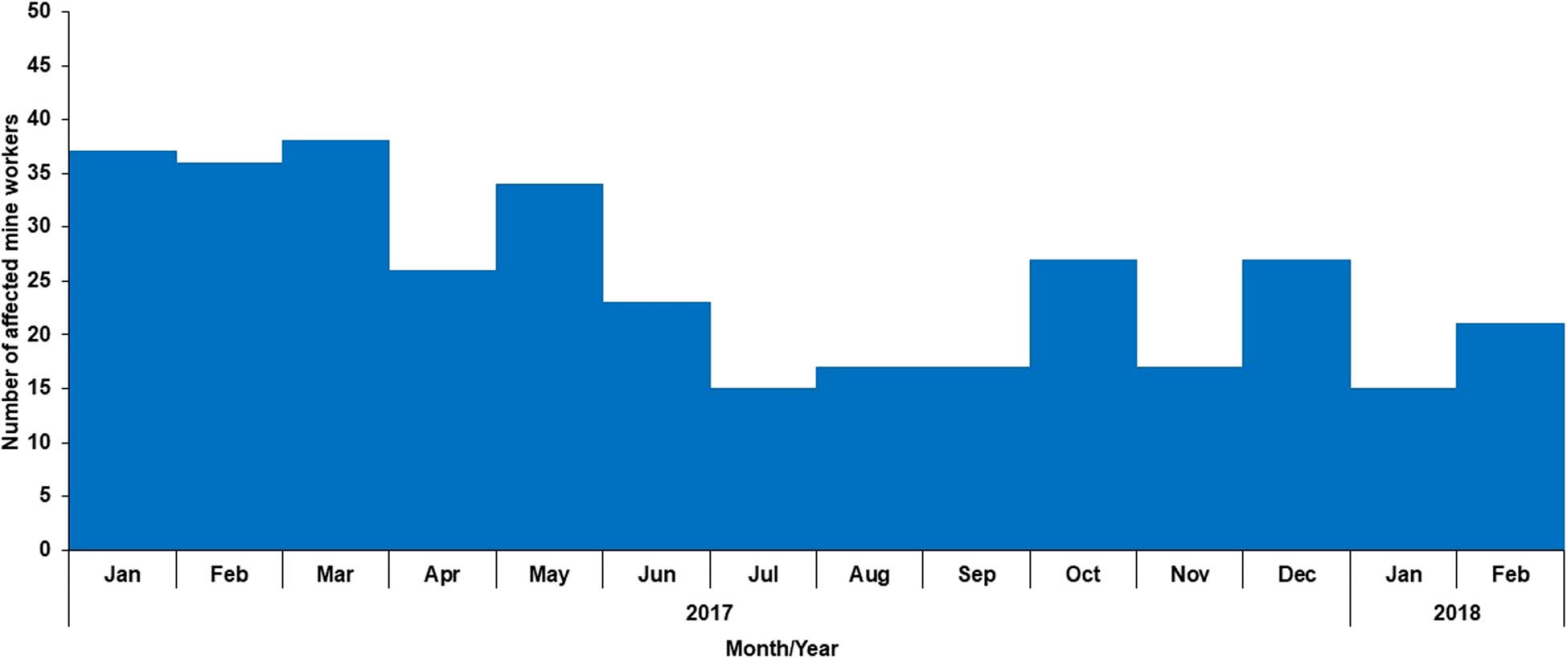
Distribution of affected gold mine workers with reported cutaneous abscesses, January 2017 to February 2018, *n* = 350

From January 2017 to February 2018, 10% (350/3582) of gold mine workers sought care for cutaneous abscesses, with higher numbers being reported in the first 6 months of 2017 (Fig. 1).

### Epidemiological findings from medical record review and face-to-face interviews

**Fig. 2:**
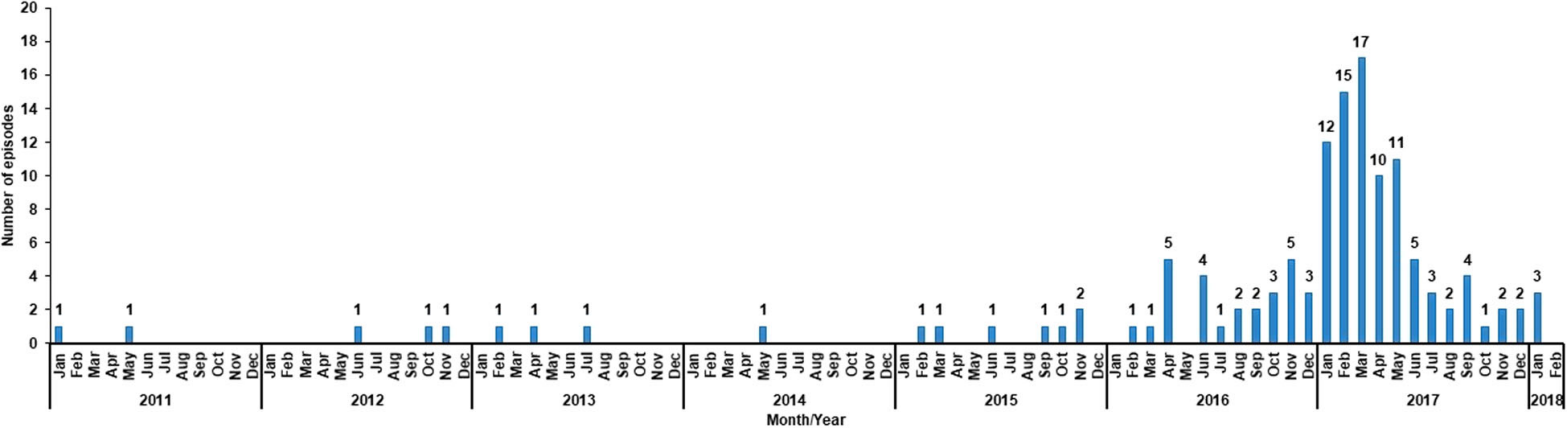
Number of episodes of cutaneous abscesses presented by 47 gold mine workers at the on-site occupational health clinic, *n* = 130 recorded episodes

Of a random sample of 50 cases, 47 medical files were available for review. The totals represent the respective available data. Among the 47 symptomatic mine workers, 96% were male (45/47) with a mean age of 43 years (SD = 7) at their last clinic visit. Sixty percent (27/45) lived off-site, while the remaining 40% (18/45) stayed in hostels located on the mine premises. Four occupational fields identified, of which 52% (24/46) were involved in stoping (ore extraction activities underground) followed by 26% (12/46) in roving underground (mine workers are not assigned to one particular level). Symptomatic mine workers operated on five levels underground, of which 28% (13/47) worked on a particular level (results not shown due to confidentiality reasons) (Table 1). Forty-two percent (18/43) were HIV-seropositive, 47% (21/45) had a history of smoking (previous or current) and 46% (21/46) had a history of alcohol consumption (previous or current) (Table 1). The 47 workers had attended the occupational clinic for treatment of multiple episodes of cutaneous abscesses (data were collected on all episodes from 14 January 2011 to 15 January 2018). As of 06 February 2018, 130 episodes of cutaneous abscesses were recorded, 21% (27/130) in 2016 and 65% (84/130) in 2017 (Fig. 2). Sixty-four percent (83/130) were recurrent episodes. Of the 60 episodes with known information, the median duration of symptoms before presentation to the occupational clinic was 3 days (IQR 2–6). The location of the cutaneous abscesses was classified into eight anatomical sites, of which 31% (41/130) were located on the lower limbs and 24% (31/130) on the upper limbs (Table 1). For episodes with a recorded treatment history, 52% (62/119) were surgically drained (incision and drainage) and 83% (101/122) were treated with at least one oral antibiotic, of whom 65% (79/122) were treated with metronidazole. Of the 24 episodes with a known recorded outcome, the median duration of resolution was 12 days (IQR 9–17) and 98% (46/47) resolved (Table 1).

**Table 1:** Demographic, medical history and clinical treatment of gold mine workers who presented with cutaneous abscesses at the on-site occupational health clinic from January through to September 2017, *n* = 47

Characteristic	Number of symptomatic mine workers (n/N^**a**^)	%
**Residence**		
Mine hostel	18/45	40
Living out	27/45	60
**Work type**		
Stoping	24/46	52
Roving underground	12/46	26
Development	5/46	11
Shaft and Services	5/46	11
**Medical history**		
Diabetes mellitus	2/45	4
Tuberculosis	2/46	4
HIV infection	18/43	42
Skin conditions	2/27	7
History of smoking	21/45	47
History of alcohol consumption	21/46	46
Other medical conditions	3/46	7
**Recurrence**		
New	47/130	36
Recurrent	83/130	64
**Site of cutaneous abscess**		
Lower limbs	41/130	31
Upper limbs	31/130	24
Hands	24/130	18
Face	16/130	12
Buttocks and perianal	7/130	5
Trunk and back	6/130	5
Groin	2/130	2
Other	2/130	2
Head and neck	1/130	1
**Treatment**		
Incision and drainage	62/119	52
Oral antibiotics^b^	101/122	83
Metronidazole	79/122	65
Amoxicillin/ampicillin	50/122	41
Cloxacillin	46/122	38
Clindamycin	14/122	12
Amoxicillin-clavulanic acid	2/122	2
Doxycycline	2/122	2
Topical antibiotics	48/120	40
Dressings	78/121	65
**Resolved (yes)**	46/47	98

^a^Denominators are less than the column total because of missing data, ^b^symptomatic mine workers often received a combination of antibiotics at the same time

Fifty-eight interviews were conducted among mine workers who were eligible and gave consent to be interviewed. Seventy-six percent (44/58) of these mine workers worked the morning shift. Fifteen of the 58 mine workers reported current or prior cutaneous abscesses, of whom 80% (12/15) were from the morning shift. We found no differences among mine workers with and without cutaneous abscesses (Table 2).

**Table 2:** Comparison of exposure characteristics of gold mine workers who presented with and without cutaneous abscesses, 21 February 2018, *n* = 58

Characteristic	Mine workers without cutaneous abscesses (***N*** = 43^**a**^)	Mine workers with cutaneous abscesses (***N*** = 15^**a**^)	***P***-value
	**n (%)**	**n (%)**	
**Sex**			0.32
Female	6 (100)	0 (0)	
Male	37 (71)	15 (29)	
**Age**			0.96
Mean age (SD)	39 (9)	39 (9)	
**Residence**			0.24
Mine hostel	6 (67)	3 (33)	
Living out	37 (77)	11 (23)	
**Work type**			0.67
Stoping	29 (73)	11 (27)	
Roving underground	1 (50)	1 (50)	
Shaft and services	2 (100)	0 (0)	
Traming	1 (100)	0 (0)	
Other	1 (50)	1 (50)	
**HIV status**			0.87
No	32 (71)	13 (29)	
Yes	7 (88)	1 (12)	
**History of smoking**			1.00
No	31 (74)	11 (26)	
Yes	12 (75)	4 (25)	
**History of alcohol consumption**			1.00
No	22 (73)	8 (27)	
Yes	21 (75)	7 (25)	
**Cuts/Scratches over the last week**			0.64
No	39 (75)	13 (25)	
Yes	4 (67)	2 (33)	
**Frequency of wearing protective clothing**			1.00
Always (100%)	39 (75)	13 (25)	
Most of the time (80%)	3 (75)	1 (25)	
**Sharing of protective clothing**			1.00
No	40 (74)	14 (26)	
Yes	1 (100)	0 (0)	
**Sets of protective clothing used in one shift**			1.00
One set	10 (77)	3 (23)	
Two sets	27 (73)	10 (27)	
More than two sets	6 (75)	2 (25)	
**Change into protective clothing**^**b**^			0.49
Mine change rooms	21 (78)	6 (22)	
Underground	11 (65)	6 (35)	
**Store protective clothing**			1.00
Mine change rooms	23 (74)	8 (26)	
Underground	9 (75)	3 (25)	
Place of stay	11 (73)	4 (27)	
**Rinse protective clothing with untreated water**			1.00
No	8 (57)	6 (43)	
Yes	7 (64)	4 (36)	
**Bring protective clothing to surface**			0.29
After every shift	31 (79)	8 (21)	
Once a week	3 (60)	2 (40)	
Twice a week	2 (100)	0 (0)	
Never	2 (50)	2 (50)	
**Daily wash of protective clothing**			0.66
No	6 (86)	1 (14)	
Yes	30 (73)	11 (27)	
**Place where protective clothing were laundered**			1.00
Place of stay	14 (74)	5 (26)	
Mine laundromat	26 (72)	10 (28)	
**Wash at mine change rooms after a shift**			1.00
No	4 (80)	1 (20)	
Yes	36 (73)	13 (27)	
**Worked in a different section of the mine**^**c**^			1.00
No	27 (73)	10 (27)	
Yes	14 (78)	4 (22)	

^a^Denominators are less than the column total because of missing data, ^b^protective clothing used during a shift, ^c^over the last 12 months

### Phenotypic characterisation of *S. aureus* isolates

From 22 November 2017 through to 13 March 2018, 29 pus aspirate specimens were received by the NICD. Of these, *S. aureus* was cultured from 86% (25/29), *S. epidermidis* from one and no bacterial growth for three. All 25 *S. aureus* isolates were susceptible to cloxacillin (based on a negative cefoxitin screen), trimethoprim/sulfamethoxazole and mupirocin. Eighty-four percent (21/25) were susceptible to clindamycin but resistant to penicillin (MIC≥8 μg/ml). Twelve percent (3/25) were susceptible to both clindamycin and penicillin, while one isolate was resistant to clindamycin (erythromycin MIC> 4 μg/ml) and penicillin (Table 3).

**Table 3:** Pus aspirates and skin scrapings submitted to the National Institute for Communicable Diseases for testing, 22 November 2017 to 13 March 2018, *n* = 35

Isolate number	Specimen type	Collection date	Organism	PVL	Cefoxitin^**b**^	Pen MIC	Pen Interp	Eryth MIC	Eryth Interp	Clinda MIC	Clinda Interp	SXT MIC	SXT Interp	Mupirocin
DRKM1	Pus aspirate	27/11/2017	*Staphylococcus aureus*	POS	NEG	> 8	R	≤0.5	S	≤0.25	S	≤2	S	S
DRKM2	Pus aspirate	23/11/2017	*Staphylococcus aureus*	POS	NEG	8	R	≤0.5	S	≤0.25	S	≤2	S	S
DRKM3	Pus aspirate	22/11/2017	*Staphylococcus aureus*	POS	NEG	> 8	R	≤0.5	S	≤0.25	S	≤2	S	S
DRKM4	Pus aspirate	22/11/2017	*Staphylococcus aureus*	POS	NEG	8	R	≤0.5	S	≤0.25	S	≤2	S	S
DRKM5	Pus aspirate	04/12/2017	*Staphylococcus aureus*	POS	NEG	8	R	≤0.5	S	≤0.25	S	≤2	S	S
DRKM6	Pus aspirate	29/11/2017	No bacterial growth	NT										
DRKM7	Pus aspirate	04/12/2017	*Staphylococcus aureus*	POS	NEG	> 8	R	≤0.5	S	≤0.25	S	≤2	S	S
DRKM8	Pus aspirate	14/12/2017	*Staphylococcus aureus*	POS	NEG	> 8	R	≤0.5	S	≤0.25	S	≤2	S	S
DRKM9	Pus aspirate	18/12/2017	*Staphylococcus aureus*	NEG	NEG	> 8	R	> 4	R	≤0.25	R	≤2	S	S
DRKM10	Pus aspirate	19/12/2017	*Staphylococcus aureus*	POS	NEG	8	R	≤0.5	S	≤0.25	S	≤2	S	S
DRKM11	Pus aspirate	20/12/2017	*Staphylococcus aureus*	POS	NEG	≤0.12	S	≤0.5	S	≤0.25	S	≤2	S	S
DRKM12	Pus aspirate	20/12/2017	*Staphylococcus aureus*	POS	NEG	8	R	≤0.5	S	≤0.25	S	≤2	S	S
DRKM13	Pus aspirate	18/01/2018	*Staphylococcus aureus*	POS	NEG	> 8	R	≤0.5	S	≤0.25	S	≤2	S	S
DRKM14	Pus aspirate	18/01/2018	No bacterial growth	NT										
DRKM15	Pus aspirate	18/01/2018	No bacterial growth	NT										
DRKM16	Pus aspirate	23/01/2018	*Staphylococcus aureus*	POS	NEG	> 8	R	≤0.5	S	≤0.25	S	≤2	S	S
DRKM17	Pus aspirate	06/02/2018	*Staphylococcus aureus*	POS	NEG	< 0.12	S	≤0.5	S	≤0.25	S	≤2	S	S
DRKM18	Pus aspirate	06/02/2018	*Staphylococcus aureus*	POS	NEG	> 8	R	≤0.5	S	≤0.25	S	≤2	S	S
DRKM19	Pus aspirate	06/02/2018	*Staphylococcus aureus*	POS	NEG	> 8	R	≤0.5	S	≤0.25	S	≤2	S	S
DRKM20	Pus aspirate	08/02/2018	*Staphylococcus epidermidis*	NT										
DRKM21	Pus aspirate	12/02/2018	*Staphylococcus aureus*	POS	NEG	8	R	≤0.5	S	≤0.25	S	≤2	S	S
DRKM22	Pus aspirate	12/02/2018	*Staphylococcus aureus*	POS	NEG	< 0.12	S	≤0.5	S	≤0.25	S	≤2	S	S
DRKM23	Pus aspirate	15/02/2018	*Staphylococcus aureus*	POS	NEG	> 8	R	≤0.5	S	≤0.25	S	≤2	S	S
DRKM30	Pus aspirate	21/02/2018	*Staphylococcus aureus*	POS	NEG	8	R	≤0.5	S	≤0.25	S	≤2	S	S
DRKM31	Pus aspirate	22/02/2018	*Staphylococcus aureus*	POS	NEG	> 8	R	≤0.5	S	≤0.25	S	≤2	S	S
DRKM32	Pus aspirate	12/03/2018	*Staphylococcus aureus*	POS	NEG	> 8	R	≤0.5	S	≤0.25	S	≤2	S	S
DRKM33	Pus aspirate	13/03/2018	*Staphylococcus aureus*	POS	NEG	> 8	R	≤0.5	S	≤0.25	S	≤2	S	S
DRKM34	Pus aspirate	13/03/2018	*Staphylococcus aureus*	POS	NEG	> 8	R	≤0.5	S	≤0.25	S	≤2	S	S
DRKM35	Pus aspirate	12/03/2018	*Staphylococcus aureus*	NEG	NEG	> 8	R	≤0.5	S	≤0.25	S	≤2	S	S
DRKM24^a^	Skin scrapings	21/02/2018	*Staphylococcus haemolyticus*	NEG	POS	8	R	> 4	R	> 2	R	> 4	R	R
DRKM25^a^	Pus aspirate	21/02/2018	*Staphylococcus aureus*	POS	NEG	> 8	R	≤0.5	S	≤0.25	S	≤2	S	S
DRKM26^a^	Skin scrapings	21/02/2018	*Staphylococcus aureus*	POS	NEG	> 8	R	≤0.5	S	≤0.25	S	≤2	S	R
DRKM27^a^	Skin scrapings	21/02/2018	*Staphylococcus aureus*	POS	NEG	> 8	R	≤0.5	S	≤0.25	S	≤2	S	S
DRKM28^a^	Pus aspirate	21/02/2018	*Staphylococcus aureus*	POS	NEG	> 8	R	≤0.5	S	≤0.25	S	≤2	S	S
DRKM29^a^	Pus aspirate	21/02/2018	*Staphylococcus aureus*	POS	NEG	> 8	R	≤0.5	S	≤0.25	S	≤2	S	S

^a^Specimens obtained from mine workers who reported to have cutaneous abscesses during interviews, ^b^Cefoxitin screen, *PVL* Panton-Valentine leukocidin, *POS* Positive, *NEG* Negative, *NT* Not tested, *MIC* Minimum inhibitory concentration, *Interp* interpretation, *Pen* Penicillin, *Eryth* Erythromycin, *Clinda* Clindamycin, *SXT* Trimethoprim/sulfamethoxazole, *S* Susceptible, *R* Resistant

Of the 15 mine workers who reported to have cutaneous abscesses during interviews, 80% (12/15) were examined by the team of medical doctors from the NICD. Six skin scrapings and three pus aspirates were obtained from eight affected mine workers. Of these nine specimens, we cultured *S. aureus* from 56% (5/9) and *S. haemolyticus* from one. Three specimens were not tested due to poor specimen quality. All five isolates displayed similar AST profiles to specimens submitted to NICD from cases. All five *S. aureus* isolates were penicillin-resistant, but susceptible to cloxacillin, trimethoprim/sulfamethoxazole and clindamycin. One of the five isolates was resistant to mupirocin (MIC> 256 μg/ml) (Table 3).

Eighty-three nasopharyngeal swabs were collected. Of these, we cultured *S. epidermidis* from 67% (56/83), *S. aureus from* 14% (12/83), *S. haemolyticus* from 10% (8/83), and other *Staphylococcus* species from 8% (7/83). Of the 83 mine workers who had nasopharyngeal swabs taken, 53% (44/83) were interviewed. Of the 15 mine workers who reported to have cutaneous abscesses, 11 had nasopharyngeal swabs taken. All 11 nasopharyngeal swabs cultured *S. epidermidis*. Of the 43 mine workers who reported not to have cutaneous abscesses, 33 had nasopharyngeal swabs taken. Five (NP6, NP7, NP16, NP17 and NP28) of the 33 nasopharyngeal swabs cultured *S. aureus*.

### Genotypic characterisation of *S. aureus* isolates

**Fig. 3:**
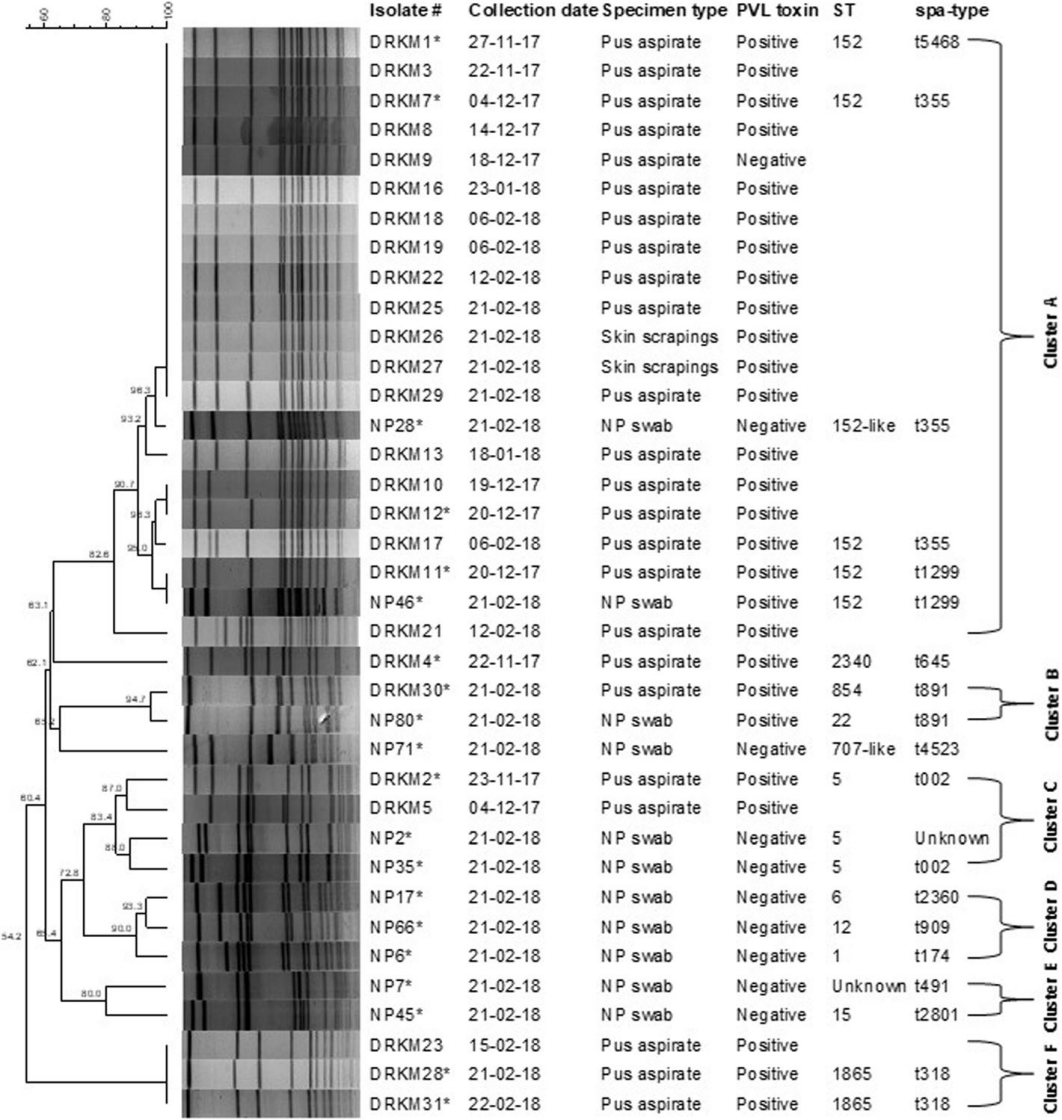
Pulsed-field gel electrophoresis DNA fingerprint patterns (*Sma*I-digestion) of *Staphylococcus aureus* isolates, 22 November 2017 through to 22 February 2018, *n* = 37. Isolates were considered genetically related if the banding patterns showed ≥80% similarity. A cluster was defined if isolates showed ≥90% similarities. *Isolates selected for whole genome sequencing analysis

Of the 42 *S. aureus* isolates (30/38 pus aspirates or skin scrapings and 12/83 nasopharyngeal swabs), 71% (30/42) were PCR-positive for the *luk*S/F-PV gene. PFGE analysis was performed on 37 *S. aureus* isolates, only isolates characterised from 22 November 2017 through to 22 February 2018 were included in the analysis. Six clusters (A-F) were identified, of which 57% (21/37) made up cluster A (isolates clustered together ≥90%). Two isolates, DRKM4 and NP71 had PFGE profiles that were distinguishable from the other isolates suggesting that these isolates were not related (Fig. 3). Based on WGS, a sequence type (ST) and *spa* type were assigned for 19 of the 20 outbreak isolates as shown in Fig. 3. Similarities in the ST and *spa* type were noted for three PFGE clusters except clusters B, D and E (Fig. 3). Six (DRKM, DRKM7, DRKM11, DRKM12, NP28 and NP46) of the 20 isolates chosen for WGS analysis belonged to ST152, all six isolates belonged to cluster A.

### Findings from bio-risk assessment

A detailed summary of the bio-risk assessment is outline in Table 4.

**Table 4:** Summary of the bio-risk assessment conducted on 21 February 2018

Parameters assessed	Existing controls in place	Risk factors
Cleaning of walls and floors	Germogon targets Gram-positive and Gram-negative bacteria Citriklenz targets Gram-positive and Gram-negative bacteria, yeast and moulds	none
Cleaning safety boots after underground shift	Vitroglo targets Gram-positive and Gram-negative bacteria, yeast and moulds	Used for sanitizing, cleaning and deodorizing sanitary ware as per manufacturers instruction
Laundering	Dirty and clean laundry segregated Presence of dedicated washing machines for male and female clothes	No laundering of towels used in change rooms
Personal Protective Equipment	Provided before each shift	Sharing of gloves between workers Workers washed protective clothing with untreated water and hung them for drying at the underground surface
Dressing of infectious wounds	Individually wrapped packs provided for each worker in the occupational health clinic	none
Waste disposal	Re-usable medical waste bins and sharps containers for disposal of biological waste Waste collected and disposed by a subcontractor	none
Hand hygiene	All consultation rooms and change rooms had dedicated soap dispensers and basins for hand washing	No soap provided underground and untreated water was used to rinse hands and cool bodies
Drinking water quality	Week 1: TPC = 14,500 cfu/ml, faecal coliforms = 1 cfu/100 ml Week 2: TPC = 6130 cfu/ml Week 3: TPC = 1018 cfu/ml, faecal coliforms = 19 cfu/100 ml	TPC were above the limits of 1000 cfu/ml, coliform counts > 10 cfu/100 ml and presence of faecal coliforms
Engineering controls	Presence of ultraviolet germicidal irradiation lamps for infection control in change rooms	Some lights were non-functional and there was no evidence of service records and maintenance stickers
Environmental factors	Temperature and humidity monitored by an occupational hygienist underground	Temperature and relative humidity (dry bulb) ranging from 30.5 °C–31.3 °C and 61.1–91.0%

*TPC* Total plate count, *cfu/ml* Colony forming units per millilitre

## Discussion

We describe a large outbreak of cutaneous abscesses among mine workers at a gold mine in Gauteng, South Africa. Skin diseases are common among South African mine workers [11]. However, they are seldom linked to a causative agent [11]. For instance, a medical record review of 507 patient files from a coal company (2005–2006) showed that 62% of skin disorders were diagnosed as infection-related [11]. In our study, findings from the medical record review showed that reports of cutaneous abscesses were identified prior to 2017, which may suggest that this outbreak had started earlier. Of the records evaluated, only one affected mine worker had a pus swab taken for testing: *S. aureus*, *Klebsiella pneumoniae* and *Enterobacter cloacae* were isolated (results not shown). Our findings showed that more than 60% of episodes were recurrent infections. It has been reported that identifying the causative pathogen may not be necessary for treating uncomplicated skin infections, but cultures can provide valuable information in patients with recurrent skin infections [12]. Our findings showed that more than 60% of episodes were treated with metronidazole. There is an Essential Drug List (EDL) at national level to guide clinicians for empirical treatment options for all organ systems including infectious causes. Each facility practitioner makes a decision based on assessment of possible cause of infection. Among our cases, mixed infections have been considered and number of antimicrobial agents were options including cover for anaerobic bacteria. Once unsuccessful with this treatment approach, NICD was consulted for investigation and assistance.

We showed that the causative agent responsible for this outbreak was PVL-producing MSSA. To our knowledge, no other outbreaks of PVL-producing MSSA SSTIs have been reported in mining facilities in South Africa. PVL is not necessarily associated with community-associated MRSA infections [13]. However, several outbreaks of skin infections due to PVL-producing MSSA have been previously described in community settings [14–16]. The presence of PVL was observed in a relatively high percentage of the MSSA isolates obtained from pus aspirates and skin scrapings (28/30 isolates were PCR-positive), but not from nasopharyngeal swabs (2/12 isolates were PCR-positive). This is consistent with findings from previous studies where the presence of PVL was low in specimens obtained from the nasopharynx [17].

PFGE and WGS analysis from this study revealed more than one cluster of *S. aureus* infections involving closely related isolates. More than half of the isolates clustered together indicating clonal spread and all six isolates chosen from cluster A for WGS belonged to ST152, making this the dominant outbreak isolate. In this study, the introduction of ST152 PVL-producing MSSA in the gold mine is still unknown. ST152 PVL-producing *S. aureus* was reported to be common in West and Central Africa [18].

High temperature and relative humidity measured at the underground level can support microbial growth, which can impact on employees’ health. Washing facilities (showers, hand wash basins) in all change rooms and the laundry was available at the gold mine. Infections can be acquired in settings where people interact through physical contact or with contaminated surfaces [19]. Although laundering practices appeared sufficient, damp towels used by employees were hanging on the lockers in the shared changing rooms during the survey and could expose workers to infectious agents. Re-used cloth towels can be contaminated with microorganisms. Sifuentes et al reported that re-usable hospital towels contained 93% viable microbes after laundering, with clean towels having the highest microbial load suggesting that the laundering practices were inadequate [20]. Employees with abscesses in particular can contribute to pathogen transmission and/or reinfection via human or surface contact. Sharing of protective clothing was evident and presents a potential source of transmission. Despite the in-house laundry service being accessible, some workers washed their protective clothing with raw water which previously yielded high TPC and faecal coliforms. Which were above the recommended limits [21]. We recommended that the administrative controls be strengthened by incorporating an awareness programme on infection prevention and control practices (IPC) for mine workers. The effectiveness of the disinfectant in reducing or inactivating the bacterial load is important in infection control [22, 23]. The bactericidal characteristics of the soap used for rinsing safety boots after shift work was not stated on the material safety data sheet except that it serves as a sanitizer and deodoriser. The dilution used was also not known during the assessment. The antibacterial performance of the disinfectant used should thus be considered and evaluated before application considering that the lower limbs were most affected. The medical hub occupational health clinic was well organised with administrative controls in place. These included separation of patients for cleaning and dressing infected sites, dedicated protective clothing, proper storage and disposal of medical waste, natural ventilation policy and maintenance of ultraviolet germicidal irradiation fixtures.

The study design was a weakness of this study; the sample size was small and could not detect differences among mine workers with and without cutaneous abscesses. Another limitation was the nasopharyngeal colonisation study, which was conducted at a single point in time for one group of mine workers only, thus excluding additional mine workers who may have been colonised.

## Conclusions

In summary, PVL-producing MSSA caused an outbreak of cutaneous abscesses among underground workers at a gold mining company. We identified isolates with more than one ST that were responsible for this outbreak. Overall, we observed poor adherence to protective clothing use, and suboptimal administrative and engineering controls. The use of disinfectants, laundering of overalls and drying of towels should be critically re-evaluated to prevent cross contamination. Worker awareness of infection prevention and control practices should be strengthened.

## Supplementary information

**Additional file 1:.** Questionnaire S1. Data collection tool.

## Data Availability

Some restrictions will apply. Whole genome sequencing data is publicly available by accessing the following links: https://www.ncbi.nlm.nih.gov/bioproject/?term=PRJNA560164 and https://www.ncbi.nlm.nih.gov/bioproject/?term=PRJNA548666. However, line patient data cannot be shared publicly due to confidentiality concerns.

## Abbreviations

AST: Antimicrobial susceptibility testing
BHI: Brain heart infusion
cfu/ml: Colony forming units per millilitre
Clinda: Clindamycin
CLSI: Clinical and Laboratory Standards Institute
EDL: Essential Drug List
Eryth: Erythromycin
Interp: Interpretation
IPC: Infection prevention and control practices
MALDI-TOF: Matrix assisted laser desorption ionization-time of flight mass spectrometer
MIC: Minimum inhibitory concentrations
MLST: Multi locus sequence typing
MSSA: Methicillin-susceptible *Staphylococcus aureus*
NEG: Negative
NHLS: National Health Laboratory Service
NICD: National Institute for Communicable Diseases
NT: Not tested
PCR: Polymerase chain reaction
Pen: Penicillin
PFGE: Pulsed-field gel electrophoresis
POS: Positive
PVL: Panton-Valentine leukocidin
R: Resistant
S: Susceptible
SSTIs: Skin and soft tissue infections
ST: Sequence type
SXT: Trimethoprim/sulfamethoxazole
TPC: Total plate count
WGS: Whole-genome sequencing
