# Supplementary material for: An outbreak of cutaneous abscesses caused by Panton-Valentine leukocidin-producing methicillin-susceptible Staphylococcus aureus among gold mine workers, South Africa, November 2017 to March 2018
Source: BMC Infect Dis. 2020 Aug 24;20:621. doi: 10.1186/s12879-020-05352-5 (PMC7446146; doi:10.1186/s12879-020-05352-5)
Supplement: Supplementary file 1 — Additional file 1:. Questionnaire S1. Data collection tool. [file 12879_2020_5352_MOESM1_ESM.pdf]

NATIONAL INSTITUTE FOR COMMUNICABLE DISEASES
Division in the National Health Laboratory Service

Employee Industry Number: ____________________

2017 SKIN ABSCESS OUTBREAK:

QUESTIONNAIRE – CROSS-SECTIONAL STUDY

Instructions

1. Read the introduction and the informed consent form to the potential participant.
2. Obtain individual's signature if they agree to participate.
3. Hand an information sheet to participants to keep with them.
4. If the individual does not agree to participate, complete the refusal section.
5. If the individual agrees to participate, determine whether the individual meets the inclusion criteria: *Any person aged 18 years or older, engaged in underground work and employed for at least one month (as of the date of interview) at the Gold Mine.*
6. For all participants who do not have a skin lesion or bump anywhere on their body, sections A-F should be completed by the interviewer
7. For all participants who have a skin lesion or bump anywhere on their body, sections A-G should be completed by the interviewer and referred to a doctor or nurse to complete sections H-I. Obtain individual's signature if they agree to have a sample taken.
8. Read out all the possible options before selecting an appropriate option.

Employee Industry Number: ____________________

Introduction

Hello. My name is ____

I work for the National Institute for Communicable Diseases (NICD). There have been mine workers who have been affected with skin lesions at the gold mine. The NICD has been requested by the management of the gold mining company to assist with an investigation.

I would like to ask you some general questions about your health and the work that you do at the mine. We will record your answers on a questionnaire. This interview will take about 20 minutes.

All employee information will be kept confidential and will only be used for the purposes of this investigation. There should be no risk if you agree to take part in the investigation.

We hope that by collecting the information we will be able to prevent further cases at the mine.

Do you agree to be interviewed?	☐ 1 Yes (obtain participant signature, continue with the interview)<br>☐ 2 No (complete the refusal section)

Participant signature: ______________________ Date: 21 February 2018

Reason for not agreeing to be interviewed	☐ 1 Not interested<br>☐ 2 Too busy<br>☐ 3 Concerned about confidentiality of information provided<br>☐ 4 Other (specify):____________________

Employee Industry Number: ___________________

SECTION A: INCLUSION CRITERIA			
**Q1**	**Q1.1** Are you aged 18 years or older?<br><br>**Q1.2** Are you engaged in underground work?<br><br>**Q1.3** Are you employed at the gold mine?<br><br>**Q1.4** Have you been working at the gold mine for at least one month?	☐ 1 Yes<br>☐ 2 No<br><br>☐ 1 Yes<br>☐ 2 No<br><br>☐ 1 Yes<br>☐ 2 No<br><br>☐ 1 Yes<br>☐ 2 No	**If Yes to all four questions (Q1.1 to Q1.4)**, skip to **Q2**<br>**If No,** end questionnaire
If the inclusion criteria are not met:	**Thank you for your time. We will not include you in the study. Goodbye.**		

SECTION B: OUTCOME STATUS			
**Q2**	**Q2.1** Do you at this moment have a skin lesion or bump anywhere on your body (head, neck, arms, or legs)	☐ 1 Yes<br>☐ 2 No	**If Yes**, continue with **Q2.2**, complete sections A-G and refer to doctor or nurse to complete sections H-I<br><br>**If No**, complete sections A-F only and end questionnaire
	**Q2.2** Where do you think that you got these skin lesion(s) or bumps on your body from?		

QUESTIONNAIRE

SECTION C: INTERVIEWER DETAILS		
**Q3**	Name and surname / initials of person completing the questionnaire:	
**Q4**	Date of interview (DD/MM/YYYY):	21/02/2018
**Q5**	Shift	☐ 1 Morning<br>☐ 2 Afternoon<br>☐ 3 Evening

Continue with SECTION D: EMPLOYEE DETAILS

Employee Industry Number: ___________________

SECTION D: EMPLOYEE DETAILS			
**Q6**	Sex (observation)	☐ 1 Male<br>☐ 2 Female	
**Q7**	What is your date of birth (DD/MM/YYYY)?<br>If the participant does not know his / her date of birth; ask how old he or she is (age in years).	☐ 1 Known _____________<br>☐ 2 Unknown<br><br>________ years	
**Q8**	Where do you stay after work?	☐ 1 Mine hostel<br>☐ 2 Living out<br>☐ 3 Unknown	
**Q9**	When did you start working at gold mine (DD/MM/YYYY)?		
**Q10**	How long have you worked at gold mine?	☐ 1 Less than 12 months<br>☐ 2 1 to 3 years<br>☐ 3 4 to 5 years<br>☐ 4 More than 5 years<br>☐ 5 Unknown	
**Q11**	What is your occupation?	☐ 1 Machine/rock drill operator<br>☐ 2 Scraper winch operator<br>☐ 3 Stope team member<br>☐ 4 Development team member<br>☐ 5 Load operator<br>☐ 6 Loco operator<br>☐ 7 Engineering assistant<br>☐ 8 Other (specify)<br>________________________<br>☐ 9 Unknown	
**Q12**	What type of work are you involved in?	☐ 1 Development<br>☐ 2 Roving surface<br>☐ 3 Roving underground<br>☐ 4 Shaft and services<br>☐ 5 Stoping<br>☐ 6 Traming<br>☐ 7 Other (specify)<br>________________________	

		☐ 8 Unknown	
**Q13**	In which section of the mine do you currently work in (number)?		
**Q14**	In which gang do you belong to?		

Continue with SECTION B: EMPLOYEE MEDICAL HISTORY

**SECTION B: EMPLOYEE MEDICAL HISTORY**			
**Q15**	Do you have any of the following medical conditions?	**Q15.1 Diabetes mellitus**<br>☐ 1 Yes ☐ 2 No<br>☐ 3 Unknown<br><br>**Q15.2 Tuberculosis (TB)**<br>☐ 1 Yes ☐ 2 No<br>☐ 3 Unknown<br>If Yes, status of TB infection<br>☐ 1 Previous ☐ 2 Current<br><br>**Q15.3 Cancer**<br>☐ 1 Yes ☐ 2 No<br>☐ 3 Unknown<br>If Yes, specify what type<br>___________________________<br><br>**Q15.4 Skin conditions (for example eczema, psoriasis)**<br>☐ 1 Yes ☐ 2 No<br>☐ 3 Unknown<br>If Yes, specify what type<br>___________________________<br><br>**Q15.5 Other medical condition**<br>☐ 1 Yes ☐ 2 No<br>☐ 3 Unknown<br>If Yes, specify what type<br>___________________________	
**Q16**	You may decide if you want to answer the next question or not.	☐ 1 Yes ☐ 2 No<br>☐ 3 Unknown	

Employee Industry Number: ___________________

	Are you HIV-positive?	If Yes, are you on antiretroviral medication?<br>☐ 1 Yes ☐ 2 No<br>☐ 3 Unknown	
**Q17**	Do you have a history of smoking	☐ 1 Yes ☐ 2 No<br>If Yes, are you a<br>☐ 1 Current smoker<br>☐ 2 Previous smoker<br>☐ 3 Unknown	If Yes, skip to **Q18**<br>If No, skip to **Q20**
**Q18**	How many cigarettes do you smoke in one day?		
**Q19**	How long have you been smoking (years)?		
**Q20**	Do you drink alcohol?	☐ 1 Yes ☐ 2 No	If Yes, skip to **Q21**<br>If No, skip to **Q24**
**Q21**	How often do you drink alcohol?	☐ 1 Everyday<br>☐ 2 Once a week<br>☐ 3 Twice a week<br>☐ 4 Three times a week<br>☐ 5 More than three times a week<br>☐ 6 Weekends only<br>☐ 7 Once a month<br>☐ 8 Other (specify)<br>________________________<br>☐ 9 Unknown	
**Q22**	What type of alcohol do you drink (beer, sorghum beer/umqombothi, cider, wine, brandy, whisky, vodka, gin, sherry, cane)?		
**Q23**	What volume of alcohol do you drink at any given time (how many cans, glasses, shots, dumpi, quart, bottles, mixed)?	Number of units: __________<br>Unit type (e.g. cans): ________<br>Volume (millilitres): _________	

Continue with SECTION F: EXPOSURE INFORMATION

**SECTION F: EXPOSURE INFORMATION**

Employee Industry Number: ___________________

**Q24**	Have you noticed any cuts or scratches on your body (arms, head, neck or legs) over the last week?	☐ 1 Yes<br>☐ 2 No<br>☐ 3 Unknown	
**Q25**	Do you wear any personal protective equipment?	☐ 1 Yes<br>☐ 2 No<br>☐ 3 Unknown	If Yes, skip to **Q26**<br>If No, skip to **Q39**
**Q26**	At any given time, do you take off the following personal protective equipment? **(tick all that apply)**	☐ 1 Overalls<br>☐ 2 Gloves<br>☐ 3 Masks<br>☐ 4 Boots<br>☐ 5 Goggles<br>☐ 6 Helmets<br>☐ 7 Knee protectors<br>☐ 8 Arm protectors<br>☐ 9 Other (specify)<br>___________________________	
**Q27**	For every shift that you have worked in the last week, how often have you worn personal protective equipment?	☐ 1 Always (100%)<br>☐ 2 Most of the time (80%)<br>☐ 3 Sometimes (50%)<br>☐ 4 Never (0%)	If options 2-4 were selected skip to **Q28**<br><br>If option 1 was selected, skip to **Q29**
**Q28**	What are the reasons for not wearing your personal protective equipment all the time?		
**Q29**	Do you share any personal protective equipment with other mine workers?	☐ 1 Yes<br>☐ 2 No<br>☐ 3 Unknown	If Yes, skip to **Q30**<br>If No, skip to **Q31**
**Q30**	Have you shared personal protective equipment with others who have or previously had skin lesions or bumps on their body?	☐ 1 Yes<br>☐ 2 No<br>☐ 3 Unknown	
**Q31**	How many sets of overalls do you use during one shift?	☐ 1 One<br>☐ 2 Two<br>☐ 3 More than two<br>☐ 4 Other (specify)<br>___________________________	If two or more than two or other, skip to **Q32**<br>If one, skip to

		☐ 5 Unknown	Q33
**Q32**	If you use more than one set of overalls, where do you change into your work overalls?	☐ 1 Mine change rooms<br>☐ 2 Underground<br>☐ 4 Other (specify)<br>_______________________<br>☐ 5 Unknown	
**Q33**	Where do you store your work overalls after a shift?	☐ 1 Mine change rooms<br>☐ 2 Underground<br>☐ 3 Where you stay<br>☐ 4 Other (specify)<br>_______________________<br>☐ 5 Unknown	If underground, skip to **Q34**<br>If not underground, skip to **Q36**
**Q34**	Do you rinse off your work overalls with water underground after a shift?	☐ 1 Yes<br>☐ 2 No<br>☐ 3 Unknown	
**Q35**	When do you bring up your work overalls from underground?	☐ 1 After every shift<br>☐ 2 Once a week<br>☐ 3 Twice a week<br>☐ 4 Never	
**Q36**	Do you wash your work overalls every day?	☐ 1 Yes<br>☐ 2 No<br>☐ 3 Unknown	If No, skip to **Q37**<br>If Yes, skip to **Q38**
**Q37**	How often do you wash your work overalls in a week?	☐ 1 Nil/zero<br>☐ 2 Once<br>☐ 3 Twice<br>☐ 4 More than two times<br>☐ 5 Unknown	
**Q38**	Where do you wash your work overalls?	☐ 1 Mine Laundromat<br>☐ 2 Where you stay<br>☐ 3 Other (specify)<br>_______________________<br>☐ 4 Unknown	
**Q39**	Do you shower/bath at the mine change rooms after a shift?	☐ 1 Yes<br>☐ 2 No<br>☐ 3 Unknown	If No, skip to **Q40**<br>If Yes, skip to **Q41**
**Q40**	Where do you shower/bath after a shift?	☐ 1 Where you stay<br>☐ 2 Other (specify)<br>_______________________	

		☐ 3 Unknown	
**Q41**	Do you share a bedroom with anyone where you stay?	☐ 1 Yes<br>☐ 2 No<br>☐ 3 Unknown	If Yes, skip to **Q42**<br>If No, skip to **Q46**
**Q42**	Do you share a bed with anyone where you stay?		
**Q43**	How many people do you share a bedroom with?		
**Q44**	What is the relationship of this person(s) with whom you share a bedroom with? **(tick all that apply)**	☐ 1 Family<br>☐ 2 Friend(s)<br>☐ 3 Co-worker(s)<br>☐ 4 Other (specify)<br>_______________________<br>☐ 5 Unknown	
**Q45**	Does anyone who you share a bedroom with have or previously had skin lesions or bumps?	☐ 1 Yes<br>☐ 2 No<br>☐ 3 Unknown	
**Q46**	Do you share a bathroom facility with anyone where you stay?	☐ 1 Yes<br>☐ 2 No<br>☐ 3 Unknown	If Yes, skip to **Q47**<br>If No, skip to **Q50**
**Q47**	How many people do you share a bathroom facility with?		
**Q48**	What is the relationship of this person(s) with whom you share a bathroom facility with? **(tick all that apply)**	☐ 1 Family<br>☐ 2 Friend(s)<br>☐ 3 Co-worker(s)<br>☐ 4 Other (specify)<br>_______________________<br>☐ 5 Unknown	
**Q49**	Does anyone who you share a bathroom facility with have or previously had skin lesions or bumps?	☐ 1 Yes<br>☐ 2 No<br>☐ 3 Unknown	
**Q50**	Have you worked in a different section(s) of the mine over the last 12 months?	☐ 1 Yes<br>☐ 2 No<br>☐ 3 Unknown	
**Q51**	Have you worked in a different gang(s) over the last 12 months?	☐ 1 Yes<br>☐ 2 No<br>☐ 3 Unknown	
**Q52**	Have you been in contact with anyone that has or previously had	☐ 1 Yes<br>☐ 2 No	If Yes, skip to **Q53**

	skin lesion(s) or bumps on their body?	☐ 3 Unknown	If No, end questionnaire if participant does not have skin lesions **OR** continue with **Q55** if the participant does have skin lesions
**Q53**	Were you in contact with this person(s) on more than one occasion?	☐ 1 Yes<br>☐ 2 No<br>☐ 3 Unknown	
**Q54**	Please explain what was the relationship of this person(s) to you?	☐ 1 Family<br>☐ 2 Friend(s)<br>☐ 3 Co-worker(s)<br>☐ 4 Other (specify)<br>_______________________<br>☐ 5 Unknown	End questionnaire if participant does not have skin lesions **OR** continue with **Q55** if the participant does have skin lesions

Continue with SECTION G: HISTORY OF SKIN LESIONS

**SECTION G: HISTORY OF SKIN LESIONS**			
**Q55**	Did you have previous episodes of skin lesions or bumps?	☐ 1 Yes<br>☐ 2 No<br>☐ 3 Unknown	If Yes, skip to **Q56**<br>If No, skip to **Q58**
**Q56**	How many episodes* of skin lesions or bumps did you have?<br><br>***A new episode is defined when the skin lesion(s) presents on a different location on the body OR after the resolution of a previous episode of skin lesion(s) within 21 days**	☐ 1 Known Number:____<br><br>☐ 2 Unknown	If Known, skip to **Q57**<br>If Unknown, skip to **Q58**
**Q57**	Date and location where skin lesions or bumps occurred<br>***Resolved is defined when the skin lesion(s) is healed OR a scar is present***	☐ 1 First episode<br>Month/Year: ___________<br>Location:_______________	

		Resolved*: ☐ 1 Yes ☐ 2 No<br>☐ 3 Unknown<br><br>☐ 2 Second episode<br>Month/Year: ___________<br>Location:_______________<br>Resolved: ☐ 1 Yes ☐ 2 No<br>☐ 3 Unknown<br><br>☐ 3 Third episode<br>Month/Year: ___________<br>Location:_______________<br>Resolved: ☐ 1 Yes ☐ 2 No<br>☐ 3 Unknown<br><br>☐ 4 Fourth episode<br>Month/Year: ___________<br>Location:_______________<br>Resolved: ☐ 1 Yes ☐ 2 No<br>☐ 3 Unknown<br><br>☐ 5 Fifth episode<br>Month/Year: ___________<br>Location:_______________<br>Resolved: ☐ 1 Yes ☐ 2 No<br>☐ 3 Unknown<br><br>☐ 6 Sixth episode<br>Month/Year: ___________<br>Location:_______________<br>Resolved: ☐ 1 Yes ☐ 2 No<br>☐ 3 Unknown<br><br>☐ 7 Seventh episode<br>Month/Year: ___________<br>Location:_______________<br>Resolved: ☐ 1 Yes ☐ 2 No<br>☐ 3 Unknown<br><br>☐ 8 Eighth episode<br>Month/Year: ___________<br>Location:_______________	

		Resolved: ☐ 1 Yes ☐ 2 No<br>☐ 3 Unknown<br><br>☐ 9 Ninth episode<br>Month/Year: ___________<br>Location:_______________<br>Resolved: ☐ 1 Yes ☐ 2 No<br>☐ 3 Unknown<br><br>☐ 10 Tenth episode<br>Month/Year: ___________<br>Location:_______________<br>Resolved: ☐ 1 Yes ☐ 2 No<br>☐ 3 Unknown	

Continue with SECTION H: CURRENT EPISODE OF SKIN LESIONS

Employee Industry Number: ___________________

Introduction

Hello. My name is ______________________________________

I am a doctor or nurse working for the National Institute for Communicable Diseases (NICD). I would like to ask you some questions on your current episode of skin lesion(s), examine current skin lesion(s) and to take a sample of the skin lesion(s) or bumps on your body to be tested at our laboratory.

This is important so that we can make a diagnosis and treat you appropriately. Your results will be shared with the doctors at the health clinic on the mine.

Taking samples of skin lesions or bumps will take about 10 minutes.

All employee information and results will be kept confidential and will only be used for the purposes of this investigation.

There might be some risk with sample collection. *Explain that the sample collection might be painful to the participant.*

**DOCTOR OR NURSE'S DETAILS**	
Name and surname or initials of person completing the form:	
Date of completion (DD/MM/YYYY):	21/02/2018
Shift	☐ 1 Morning<br>☐ 2 Afternoon<br>☐ 3 Evening

Employee Industry Number: ____________________

SECTION H: CURRENT EPISODE OF SKIN LESIONS			
**Q58**	When did the skin lesion(s) occur on your body?<br><br>If the participant does not know when the skin lesion(s) occurred; ask how many days/week(s) ago did he notice the skin lesion(s).	Date (DD/MM/YYYY): ___________________<br><br>________ day(s)<br><br>________ week(s)*<br>***One week is equivalent to seven days***	
**Q59**	Location of skin lesions **(tick all that apply)**	☐ 1 Face<br>☐ 2 Hands<br>☐ 3 Fingers<br>☐ 4 Groin<br>☐ 5 Axillae<br>☐ 6 Head and neck<br>☐ 7 Upper limbs<br>☐ 8 Trunk and back<br>☐ 9 Lower limbs<br>☐ 10 Other (specify)<br>____________________	
**Q60**	Description of skin lesion(s) **(tick all that apply)**	☐ 1 Boils<br>☐ 2 Furuncles<br>☐ 3 Superficial abscesses<br>☐ 4 Subcutaneous abscesses<br>☐ 5 Ulcers<br>☐ 6 Infected lacerations<br>☐ 7 Nodules on extremities<br>☐ 8 Cellulitis<br>☐ 9 Other (specify)<br>____________________	
**Q61**	Other description of skin lesion(s) on presentation **(tick all that apply)**	☐ 1 Painful<br>☐ 2 Swollen<br>☐ 3 Skin irritation<br>☐ 4 Itching	

		☐ 5 Pus present<br>☐ 6 Other (specify)<br>___________________	
**Q62**	Did you attend the mine clinic for treatment? **(tick all that apply)**	☐ 1 Yes ☐ 2 No<br>☐ 3 Unknown<br><br>Did the participant seek health care elsewhere?<br>☐ 1 Yes ☐ 2 No ☐ 3 Unknown<br>If Yes, specify where:<br><br>☐ 1 Local clinic<br>☐ 2 Pharmacy<br>☐ 3 Traditional healer<br>☐ 4 Relative<br>☐ 5 Friend<br>☐ 6 Other (specify)<br>___________________<br>☐ 7 Unknown	
**Q63**	Have you had treatment with antibiotics?	☐ 1 Yes ☐ 2 No ☐ 3 Unknown<br>If Yes, specify which antibiotics:<br><br>________________________<br>________________________	
**Q64**	Have you had treatment with incision and drainage?	☐ 1 Yes ☐ 2 No ☐ 3 Unknown<br>If Yes, specify the date (DD/MM/YYYY):<br>________________________<br>________________________	

Continue with SECTION I: SAMPLE COLLECTION

Employee Industry Number: ___________________

Can we to take a sample of the skin lesion or bump to be tested at our laboratory?	☐ 1 Yes (continue with sample collection)<br>☐ 2 No
Can we take a picture of the skin lesion or bump to be used for the investigation?	☐ 1 Yes (continue with taking a picture. Save the image using the employee industry number.)<br>☐ 2 No
Participant signature: _____________________	Date: 21 February 2018

**Q65**	**Sample collected:**	☐ 1 Yes ☐ 2 No<br>If No, specify the reason:<br>___________________________________	
**Sample number**	**Site of skin lesion**	**Sample type**	**Comments**
		☐ 1 Pus aspirate<br>☐ 2 Skin scrapings<br>☐ 3 Other (specify)<br>__________________	
		☐ 1 Pus aspirate<br>☐ 2 Skin scrapings<br>☐ 3 Other (specify)<br>__________________	
		☐ 1 Pus aspirate<br>☐ 2 Skin scrapings<br>☐ 3 Other (specify)<br>__________________	

End of questionnaire

Thank you for your participation
